# Insight into the Mode of Action of 8-Hydroxyquinoline-Based Blockers on the Histamine Receptor 2

**DOI:** 10.3390/bios13060571

**Published:** 2023-05-23

**Authors:** Amisha Patel, Paola L. Marquez-Gomez, Lily R. Torp, Lily Gao, Pamela Peralta-Yahya

**Affiliations:** 1 School of Chemical & Biomolecular Engineering, Georgia Institute of Technology, Atlanta, GA 30332, USA; 2 Bioengineering Graduate Program, Georgia Institute of Technology, Atlanta, GA 30332, USA; 3 School of Chemistry and Biochemistry, Georgia Institute of Technology, Atlanta, GA 30332, USA

**Keywords:** GPCRs, histamine H2 receptor, HR_H2_ blockers

## Abstract

Histamine receptor 2 (HR_H2_) blockers are used to treat peptic ulcers and gastric reflux. Chlorquinaldol and chloroxine, which contain an 8-hydroxyquinoline (8HQ) core, have recently been identified as blocking HR_H2_. To gain insight into the mode of action of 8HQ-based blockers, here, we leverage an HR_H2_-based sensor in yeast to evaluate the role of key residues in the HR_H2_ active site on histamine and 8HQ-based blocker binding. We find that the HR_H2_ mutations D98A, F254A, Y182A, and Y250A render the receptor inactive in the presence of histamine, while HR_H2_:D186A and HR_H2_:T190A retain residual activity. Based on molecular docking studies, this outcome correlates with the ability of the pharmacologically relevant histamine tautomers to interact with D98 via the charged amine. Docking studies also suggest that, unlike established HR_H2_ blockers that interact with both ends of the HR_H2_ binding site, 8HQ-based blockers interact with only one end, either the end framed by D98/Y250 or T190/D186. Experimentally, we find that chlorquinaldol and chloroxine still inactivate HR_H2_:D186A by shifting their engagement from D98 to Y250 in the case of chlorquinaldol and D186 to Y182 in the case of chloroxine. Importantly, the tyrosine interactions are supported by the intramolecular hydrogen bonding of the 8HQ-based blockers. The insight gained in this work will aid in the development of improved HR_H2_ therapeutics. More generally, this work demonstrates that Gprotein-coupled receptor (GPCR)-based sensors in yeast can help elucidate the mode of action of novel ligands for GPCRs, a family of receptors that bind 30% of FDA therapeutics.

## 1. Introduction

Histamine receptors belong to the aminergic family of Gprotein-coupled receptors (GPCRs), which includes the serotonin, muscarinic, acetylcholine, adrenergic, and dopamine receptors. Histamine receptors are involved in a variety of cellular processes, from allergies and gastric acid secretion to neurotransmission and immunomodulation [1]. There are four histamine receptor subtypes. Histamine receptor 1, HR_H1_, is involved in allergic responses and has been extensively characterized both structurally [2,3] and experimentally [4]. Histamine receptor 2, HR_H2,_ is involved in gastric acid secretion and gastroesophageal reflux diseases (GERD) [5]. Histamine receptor 3, HR_H3_, is expressed in the central nervous system and participates in neuronal histamine turnover as well as the modulation of the release of other neurotransmitters, such as dopamine and serotonin [6]. Histamine receptor 4, HR_H4_, is expressed in peripheral immune cells and is involved in immunomodulation [7]. 

Over the counter HR_H2_ blockers, such as cimetidine (Tagamet^®^) and famotidine (Pepcid^®^), are used to reduce gastric acid secretion and treat peptic ulcers and acid reflux. The chemical structure of some of these blockers, such as ranitidine (Zantac^®^), contains a tertiary amine that decomposes into the human carcinogen N-nitrosodimethylamine (NDMA) [8], which has led to their recall from the market [9]. Recently, the 8-hydroxyquinoline (8HQ)-containing compounds, chlorquinaldol and chloroxine, have been shown to block HR_H2_ activity in mammalian cells [10]. Importantly, 8HQ-based blockers lack the tertiary ammine that decomposes into NDMA, making them a promising starting point for next-generation HR_H2_ therapeutics. 

Interestingly, 8HQ-based blockers lack the positively charged amine commonly present in HR_H2_ blockers. Limited mutagenesis studies of the HR_H2_ binding site hamper our ability to understand the mode of action of 8HQ-based blockers. Here, we leverage an HR_H2_-based sensor in yeast to evaluate the role of key residues in the HR_H2_ active site on histamine and 8HQ-based blocker binding. First, using molecular docking, we identify the HR_H2_ residues involved in the binding of therapeutically relevant histamine tautomers and the 8HQ-based blockers. Then, we computationally and experimentally perform an alanine scanning of the HR_H2_ binding site and find that only HR_H2_:D186A and HR_H2_:T190A have residual activity in the presence of histamine. Finally, we determine that 8HQ-based blockers can still inactivate HR_H2_:D186A by swapping their interaction from D98 to Y250 in the case of chlorquinaldol and D186 to Y182 in the case of chloroxine. The internal stabilization of chlorinated 8HQ-based blockers, where the proton in the hydroxy group interacts with the nitrogen lone pair, is pivotal for interactions with tyrosine. Taken together, this work expands our understanding of histamine and 8HQ-blocker binding to HR_H2_, and provides evidence that GPCR-based sensors in yeast have utility in elucidating the mode of action of novel ligands for GPCRs.

## 2. Materials and Methods

***Materials*.** Luciferase expression was assayed using the NanoGlo^®^ Luciferase Assay System (Promega N1120). Histamine dihydrochloride (Sigma H7250), chlorquinaldol (Selleck S4192), and chloroxine (Selleck S1839) were purchased from the specified vendors. The HR_H2_ point mutants (HR_H2_: D98A, HR_H2_: Y182A, HR_H2_: D186A, HR_H2_: T190A, HR_H2_: Y250A, and HR_H2_: F254A) were codon optimized for *Saccharomyces cerevisiae* and commercially synthesized. Tables of plasmids (Supplementary Table S1), strains (Supplementary Table S2), and primers (Supplementary Table S3), as well as HR_H2_ mutant sequences can be found in the supplementary materials.

***Docking of 8HQ-based blockers to HR_H2_ wild type and mutants***. Histamine (ZINC388081), chlorquinaldol (ZINC119403), and chloroxine (ZINC1131) [11] were docked to the AlphaFold structure of HR_H2_ (Alphafold: P25021) [12,13]. Computational alanine scanning of HR_H2_ was conducted using the PyMOL mutagenesis wizard, with P25021 as the template. Hydrogens were introduced to the ligands using CACTUS (https://cactus.nci.nih.gov/translate/ (accessed on 27 February 2023). The HR_H2_ binding site was defined by D98, D186, and T190 [14]. Ligands were docked using AutoDock 4.2.6. Results were visualized in AutoDockTools 1.5.7 [15] and PyMOL. 

***HR_H2_ mutant construction***. HR_H2_:D98A, HR_H2_:Y182A, HR_H2_:D186A, HR_H2_:T190A, HR_H2_:Y250A, and HR_H2_:F254A were cloned into pESC-HIS3-P_TEF_-P_ADH_ [16] between *Bam*HI/*Sac*II via Gibson assembly to generate pESC-HIS3-P_TEF_-HR_H2__D98A (pPM43), pESC-HIS3-P_TEF_-HR_H2__Y182A (pPM50), pESC-HIS3-P_TEF_-HR_H2__D186A (pPM52), pESC-HIS3-P_TEF_-HR_H2__T190A (pPM53), pESC-HIS3-P_TEF_-HR_H2__Y250A (pPM54), and pESC-HIS3-P_TEF_-HR_H2__F254A (pPM49), respectively. Constructs were sequence-verified using primers LT62/LT63. 

**Figure 1:**
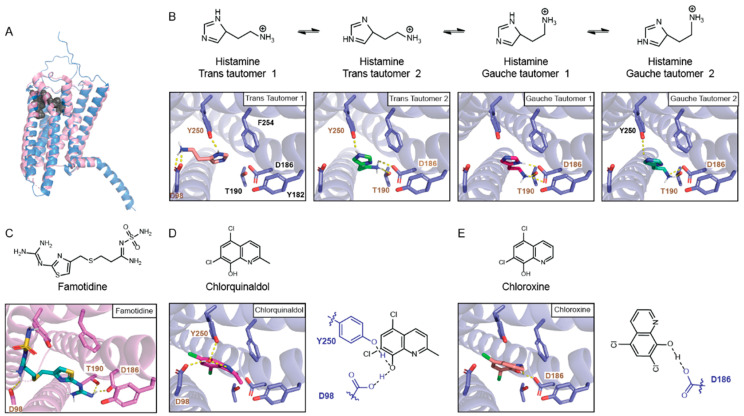
Molecular docking of histamine and 8-hydroxyquinoline-based blockers to histamine receptor 2 (HR_H2_). (**A**) Topological view of HR_H2_. Blue: HR_H2_ AlphaFold model (P25021). Residues R326-R359 do not form a secondary structure and are not shown. Pink: HR_H2_ Cryo-EM structure (PDB: 7UL3). Grey spheres: HR_H2_ active site with residues D98, D186, T190, Y182, F254, and Y250 highlighted. (**B**) Histamine tautomers docked to HR_H2_ AlphaFold model. Yellow: Electrostatic interactions (≤3 Å). Brown: Residue interacting with the ligands. (**C**) HR_H2_Cryo-EM structure bound to famotidine (PDB: 7UL3) [19]. (**D**) Docking of 8HQ-based blocker chlorquinaldol to the HR_H2_ AlphaFold model and schematic of interacting residues. (**E**) Docking of 8HQ-based blocker chloroxine to the HR_H2_ AlphaFold model and schematic of interacting residues.

***HR_H2_ mutant-based yeast sensors.*** PPY140 (*S. cerevisiae* W303 Δfar1, Δste2, Δsst2) [16] was co-transformed with pRS415-Leu2-P_FIG1_-NanoLuc [17] and either pPM43, pPM50, pPM52, pPM53, pPM54, or pPM49 to generate PPY2370, PPY2393, PPY2390, PPY2391, PPY2394, and PPY2392, respectively. To generate the no-receptor control, PPY140 was co-transformed with pRS415-Leu2-P_FIG1_-NanoLuc and pESC-HIS3-P_TEF_-P_ADH_ (PPY1809).

***Histamine sensing***. An overnight culture of PPY2370, PPY2393, PPY2390, PPY2391, PPY2394, or PPY2392 was used to inoculate 50 mL of synthetic complete medium with 2% glucose lacking histidine and leucine (SD(HL^−^)) to an OD600 = 0.06. After 18 h at 15 °C (150 rpm), the cultures were centrifuged (3500 rpm, 10 min), and resuspended in SD(HL^−^) to an OD600 = 1. In a white, flat-bottomed, 96-well plate, 190 μL pH = 7 SD (HL^−^), 8 μL of cells, and 2 μL of histamine (final concentration 10^−2^–10^4^ μM), or DMSO as a control were added. After chemical incubation (2.5 h, 30 °C, 250 rpm), 20 μL of 1:100 mixture of NanoLuc substrate to NanoLuc buffer were added, and the reaction was incubated for 30 min (30 °C, 250 rpm). Luminescence was read in a Biotek Synergy 2 using the default settings.

***HR_H2_ blocker sensing.*** The histamine sensing protocol was followed except as described. In a white, flat-bottomed, 96-well plate, 188 µL pH = 7 SD (HL^−^), 8 µL of cells, 2 µL of histamine (1 mM final concentration), and 2 µL chlorquinaldol or chloroxine (final concentration 10^−3^–10 μM) were added. For the no chemical control, no histamine or blocker was added, only 4 µL of DMSO. The no-receptor control strain was tested under the same conditions as the HR_H2_ mutant sensor strains.

## 3. Results and Discussion

**Molecular docking of HR_H2_ to histamine.** The topological view of HR_H2_ highlighting its active site is seen in Figure 1A. Early on, it was proposed that histamine’s positively charged amine interacts with D98, and that the imidazole ring tautomerizes to interact with both D186 and T190 [18]. Experimental studies in mammalian cells confirmed the necessity for D98, as the activation of HR_H2_:D98A with histamine resulted in no cAMP accumulation [14]. Positions 190 and 186 do not seem as critical to histamine binding, as the activation of HR_H2_:D186A and HR_H2_:T190A with histamine resulted in 50% and 17% of wild-type HR_H2_ activation, respectively [14]. 

To understand the binding mode of histamine to HR_H2_, we docked histamine to the AlphaFold structure of HR_H2_. Notably, although the Cryo-EM structure of HR_H2_ has been recently elucidated (PDB: 7UL3) [19], it is in the inactive state. Additionally, although the 3 Å resolution provides a good overall view of the receptor, the sidechain location is not precise. 

At pH 7.4, histamine is protonated and present in four tautomer states. The trans tautomers are the predominant form, with the gauche tautomers representing 25% of the population [20]. As shown in Figure 1B, the histamine trans tautomer 1 (TT1) makes two electrostatic interactions with D98 via the charged amine and with Y250 via the protonated nitrogen in the imidazole ring. In contrast, histamine trans tautomer 2 (TT2) interacts with D186 and T190 via the charged amine and, similarly to histamine TT1, with Y250 via the protonated nitrogen in the imidazole ring. Both histamine gauche tautomers (GTs) interact with T190 and D186, with GT2 additionally interacting with Y250 via the protonated nitrogen in the imidazole ring. Taken together, between all tautomers, histamine interacts with both sides of the binding pocket, the one described by D98/Y250 and the one described by D186/T190. Within the major tautomers, histamine TT1 engages D98 in transmembrane 2 and Y250 in transmembrane 6 (TM6) while histamine TT2 engages D186/T190 in transmembrane 5 and Y250 in TM6. 

**Molecular docking of 8HQ-based blockers to HR_H2_.** Generally, HR_H2_ blockers carry a positively charged amine that interacts with D98 and are long enough to interact with both ends of the HR_H2_ binding site. Such is the case for famotidine, whose sulfone end interacts with D98 and Y250 and guanidine group interacts with T190 and D186 [19] (Figure 1C). As shown in Figure 1D, 8HQ-based blockers interact with only one side of the HR_H2_ binding pocket. Chlorquinaldol interacts with D98 and Y250 via the hydroxyl group of the hydroxyquinoline ring (Figure 1D). Chloroxine interacts with D186 with the same moiety (Figure 1E). 

**Figure 2:**
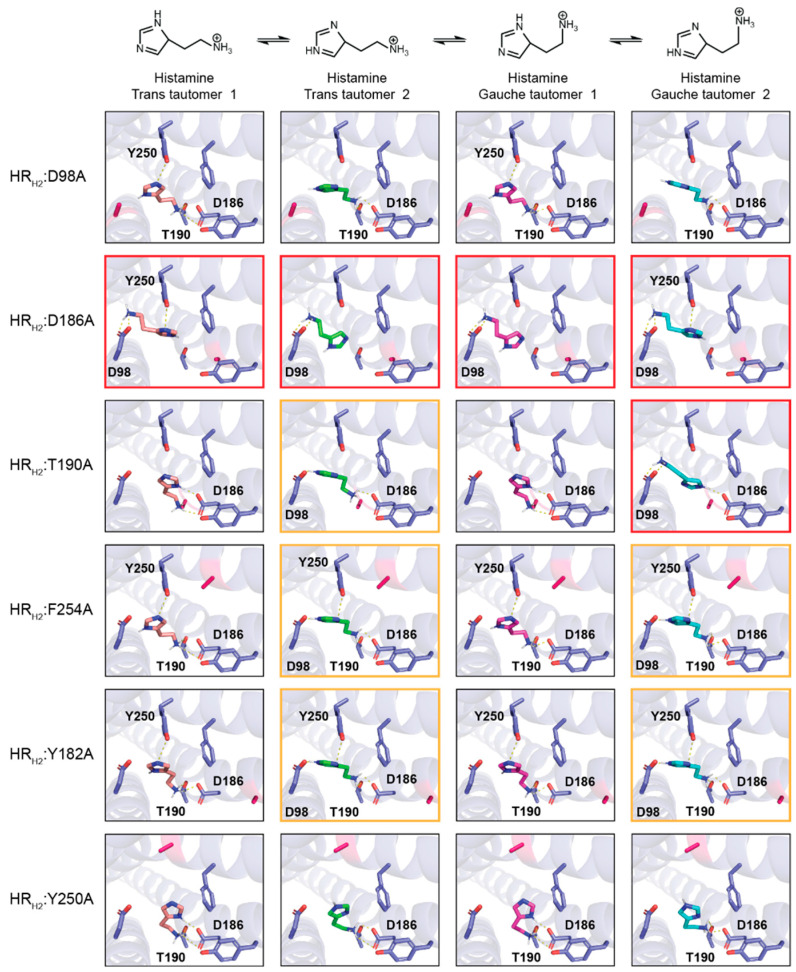
**Docking of histamine tautomers to the HR_H2_ alanine mutants.** Residues making interactions with ligands are labeled in black. Red boxes: Histamine tautomers interacting with D98 via the protonated amine. Orange boxes: histamine tautomers interacting with D98 via the protonated nitrogen in the imidazole ring.

**Virtual alanine scanning of the HR_H2_ binding site and its effect on histamine binding**. We computationally determined residues within 5 Å of histamine—D98 (D^3×32^), Y182 (Y^5×39^), D186 (D^5×3^), T190 (T^5×461^), Y250 (Y^6×51^), and F254 (F^6×55^)—and mutated them to alanine. As shown in Figure 2, the mutation of these residues to alanine significantly changes the interaction between the different histamine tautomers and HR_H2_. 

None of the histamine tautomers interact with any of the HR_H2_ mutants in a similar fashion to histamine TT1 interacting with wild-type HR_H2_. Specifically, we do not see the histamine tautomers using two electrostatic interactions via the protonated amine to engage D98 and using the protonated amine in the imidazole ring to interact with Y250. We do observe that in HR_H2_:D186A all histamine tautomers engage D98 via two electrostatic interactions using the charged amine. The only other instance where D98 is engaged via the charged amine is in HR_H2_:T190A with histamine GT2. In five HR_H2_ mutants, D98 is engaged via a single electrostatic interaction with the protonated nitrogen in the imidazole ring, specifically histamine TT2 with HR_H2_:T190A, HR_H2_:F254A, and HR_H2_:Y182A and histamine GT2 with HR_H2_:F254A and HR_H2_:Y182A. Of note, in no HR_H2_ mutant are histamine tautomers able to interact with Y250 via the protonated nitrogen in the imidazole ring. Taken together, the engagement of D98 is possible in some of the single-point mutants, specifically HR_H2_:D186A > HR_H2_:T190A > HR_H2_:F254A = HR_H2_:Y182A. In none of the HR_H2_ mutants do the histamine tautomers engage Y250 via the protonated amine, as seen in histamine TT1 with wild-type HR_H2_.

**Figure 3:**
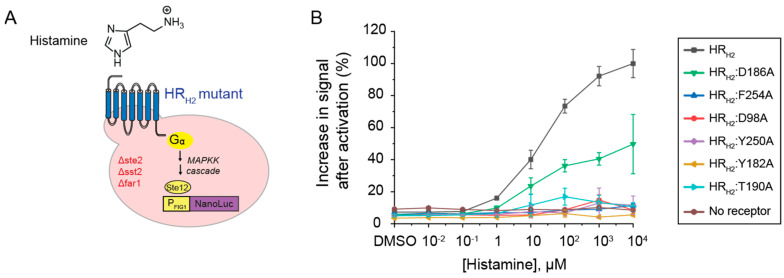
**Activation of HR_H2_ active site mutants with histamine via GPCR**−**based sensors in yeast.** (**A**) Schematic representation of the HR_H2_-based sensor. An HR_H2_ mutant is expressed on the yeast cell surface (blue). In the presence of histamine, HR_H2_ activates the MAP kinase cascade (MAPPK, yellow), ultimately resulting in the expression of a luminescence reporter gene (NanoLuc, purple). (**B**) Percent increase in signal after the activation of HR_H2_ and HR_H2_ mutants in the presence of histamine. All experiments were performed in triplicate. Shown are the means and standard deviation.

**Experimental alanine scanning of the HR_H2_ active site and its impact on histamine-driven activation**. We constructed the six HR_H2_ alanine mutants—HR_H2_:D98A, HR_H2_:D186A, HR_H2_:T190A, HR_H2_:F254A, HR_H2_:Y182A, HR_H2_:Y250A—and expressed them in a previously developed GPCR-based sensor strain that links GPCR activation to cell luminescence (Figure 3A) [17].

As shown in Figure 3B, all mutants, except for HR_H2_:D186A and HR_H2_:T190A, lost the ability to be activated by histamine. These results are consistent with previous experimental work in mammalian cells [14]. Specifically, HR_H2_:D186A retained 50% of HR_H2_ wild-type activity in the presence of histamine. This is consistent with the computationally predicted engagement of all histamine tautomers with D98 with the protonated amine (Figure 2). The 50% reduction in signal after activation could be attributed to the lack of proper engagement of Y250 via the protonated nitrogen in the imidazole ring. HR_H2_:T190A resulted in an 83% decrease in histamine activation in the yeast system. This result is consistent with the computationally predicted engagement of D98 by histamine GT2 via the protonated amine and by histamine TT2 via the protonated imidazole ring (Figure 2). The additional reduction in signal observed in HR_H2_:T190A vs. HR_H2_:D186A could be attributed to the lack of proper engagement of Y250 and the reduced number of histamine tautomers that could engage D98 via double electrostatic interactions with the protonated amine. Of note, in mammalian cells, HR_H2_:T190A results in a 50% reduction in cAMP accumulation when compared to wild-type HR_H2_ [14]. The difference could be attributed to the signaling strength of HR_H2_ in mammalian versus yeast cells.

**Figure 4:**
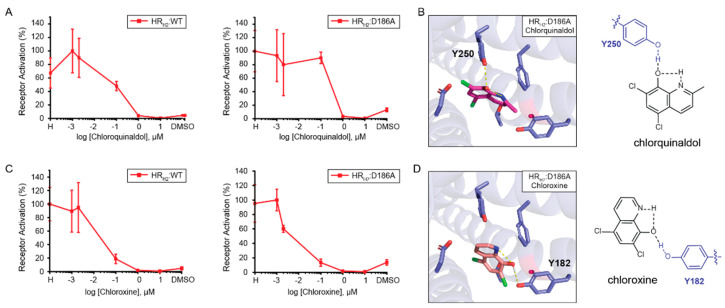
**Assessment of 8HQ**−**based blocker activity using GPCR-based sensors in yeast.** (**A**) Wild-type HR_H2_ (left) and HR_H2_:D186A (right) activation with 1 mM histamine and blocking with increasing concentrations of chlorquinaldol. (**B**) Docking of chlorquinaldol to HR_H2_:D186A and schematic of the interactions observed. (**C**) Wild-type HR_H2_ (left) and HR_H2_:D186A (right) activation with 1 mM histamine and blocking with increasing concentrations of chloroxine. (**D**) Docking of chloroxine to HR_H2_:D186A and schematic of the interactions observed. All experiments were performed in triplicate. Shown are the means and standard deviation.

**Assessing the role of position D186 in 8HQ-based blocker binding.** Given that HR_H2_:D186A results in a measurable increase in signal after activation in the presence of histamine, we assessed the role of position D186 in the binding of 8HQ-based blockers. As shown in Figure 4A, both chlorquinaldol and chloroxine block the signal after the activation of wild-type HR_H2_ and HR_H2_:D186A in the presence of histamine in a similar fashion. Docking of chlorquinaldol to HR_H2_:D186A shows chlorquinaldol intramolecular stabilization and interaction with Y250 (Figure 4B). In chlorquinaldol, the proton in the hydroxy group is highly acidic due to the two electron withdrawing chlorines on the phenyl ring. This makes the proton likely to interact with the nitrogen lone pair in the pyridine ring. The oxygen in the hydroxy group is also able to interact with the proton of the hydroxyl group of Y250. Docking of chloroxine to HR_H2_:D186A reveals a similar strategy, with chloroxine being internally stabilized and interacting with Y182 (Figure 4D). In conclusion, the intramolecular stabilization of 8HQ-based blockers is pivotal in enabling its interaction with the tyrosine residues in HR_H2_:D186A. Importantly, 8HQ-based blockers do not need to interact with D98 to inactivate the receptor. Chlorquinaldol interaction with Y250 is sufficient to inactivate HR_H2_:D186A, and chloroxine interaction with D186 is exchanged for an interaction with Y182 in order to inactivate the receptor. 

## 4. Conclusions

GPCRs are targeted by 30% of FDA-approved drugs [21]. GPCR-based sensors in yeast that link GPCR activation to reporter gene transcription can be used to gain valuable insight into the structure–activity relationship of human GPCRs. In this work, we use a HR_H2_-based sensor to elucidate the extent to which residues D98, D186, F254, Y182, and Y250 are necessary for HR_H2_ activation with histamine. We find residual activation in HR_H2_:D186A and HR_H2_:T190A, which docking studies suggest is due to the engagement of D98 via the protonated amine by some histamine tautomers. Importantly, the 50% reduction in signal activation seen in HR_H2_:D186A can be attributed to a lack of engagement of Y250. With respect to 8HQ-based blockers, we find that they engage only one end of the HR_H2_ binding site, either the end described by D98/Y250 or T190/D186. In blocking HR_H2_:D186A, intramolecular stabilization of the 8HQ-based blockers aids in the interaction with Y250 (chlorquinaldol) or Y182 (chloroxine). The results described in this work should aid in our understanding of the mode of action of 8HQ-based blockers and pave the way for the development of improved HR_H2_ blockers. More generally, this work demonstrates that GPCR-based sensors in yeast are valuable in illuminating the mode of action of novel GPCR ligands. 

## Data Availability

All the data generated or analyzed during this study is included in the published article and its supplementary materials.

## Supplementary Materials

The following supporting information can be downloaded at: https://www.mdpi.com/article/10.3390/bios13060571/s1. Table S1: Table of plasmids; Table S2: Table of strains; Table S3: Table of primers. HR_H2_ mutant sequences. References [10,16,17] are sited in the supplementary materials.

## Abbreviations

8HQ	8-hydroxyquinoline
cAMP	cyclic AMP
GPCR	Gprotein-coupled receptor
HR_H2_	histamine receptor 2
