# Supplementary material for: Insight into the Mode of Action of 8-Hydroxyquinoline-Based Blockers on the Histamine Receptor 2
Source: Biosensors (Basel). 2023 May 23;13(6):571. doi: 10.3390/bios13060571 (PMC10295836; doi:10.3390/bios13060571)
Supplement: Supplementary file 1 [file biosensors-13-00571-s001.zip › biosensors-2321722-supplementary.pdf]

Insight into the mode of action of 8-hydroxyquinoline-based blockers on the histamine receptor 2

Amisha Patel[1,2], Paola L. Marquez-Gomez[3], Lily Torp[3], Lily Gao[3], Pamela Peralta-Yahya[1,2,3*]

Supplementary Information

Table of Contents

	Page
**Supplementary Table 1:** Table of Plasmids	SI2
**Supplementary Table 2:** Table of Strains	SI2
**Supplementary Table 3:** Table of Primers	SI2
**Sequences**	SI3
**References**	SI5

Supplementary Table S1: Table of Plasmids

**Plasmid Number**	**Plasmid Name**	**Description**	**Citation**
PPY111	pKM111	pESC-His3-pTEF1-tCYC1	1
PPY1740	pEY15	pRS415-Leu2-pFIG1-NanoLuc	2
PPY2103	pRLH16	pESC-HIS3-pTEF-HR_{H2}	3
PPY2359	pPM43	pESC-His3-pADH-pTEF-HRH2_D98A	This work
PPY2366	pPM49	pESC-His3-pADH-pTEF-HRH2_F254A	This work
PPY2367	pPM52	pESC-His3-pADH-pTEF-HRH2_D186A	This work
PPY2368	pPM54	pESC-His3-pADH-pTEF-HRH2_Y250A	This work
PPY2371	pPM50	pESC-His3-pADH-pTEF-HRH2_Y182A	This work
PPY2373	pPM53	pESC-His3-pADH-pTEF-HRH2_T190A	This work

Supplementary Table S2: Table of Strains

**Strain Number**	**Description**	**Citation**
PPY140	*S. cerevisiae* W303MATa ade2-1 ura3-1 his3-11 trp1-1 leu2-3 leu2-112 can1-100 Δ*far1* Δ*ste2* Δ*sst2*	1
PPY1809	PPY140 transformed with pKM111 and pEY15	2
PPY2171	PPY140 transformed with pRLH16 and pEY15	3
PPY2389	PPY140 transformed with pPM43 and pEY15	This work
PPY2392	PPY140 transformed with pPM49 and pEY15	This work
PPY2390	PPY140 transformed with pPM52 and pEY15	This work
PPY2394	PPY140 transformed with pPM54 and pEY15	This work
PPY2393	PPY140 transformed with pPM50 and pEY15	This work
PPY2391	PPY140 transformed with pPM53 and pEY15	This work

Supplementary Table S3: Table of Primers

Primer Name	Sequence
LT62	gtcttcaatttctcaagt
LT63	ttgaaatataaataacgttc

Sequences:

> Human Histamine Receptor 2 D98A – HR_{H2} D98A

ATGGCACCTAACGGTACTGCATCTAGCTTCTGCTTAGATTCTACTGCTTGTAAGAT
TACTATTACTGTAGTATTAGCTGTATTGATTCTAATTACCGTAGCTGGCAACGTAGT
AGTATGCTTGGCTGTAGGCTTGAACAGAAGATTGCGCAACTTGACCAACTGCTTTA
TTGTTAGCTTAGCGATTACCGATCTACTACTAGGCCTACTGGTTCTACCGTTTAGC
GCTATTTATCAGTTATCATGCAAATGGAGCTTTGGTAAAGTGTTCTGCAACATTTAT
ACTTCTCTAGCTGTGATGTTGTGCACCGCTTCAATTCTGAACCTGTTTATGATTAG
CTTAGATCGCTATTGCGCGGTAATGGACCCATTACGCTATCCAGTGTTAGTGACTC
CTGTTAGAGTTGCGATTAGCCTGGTGCTGATTTGGGTGATTAGCATTACCCTGAG
CTTTCTGAGCATTCATCTGGGATGGAATAGCCGCAACGAAACCAGCAAAGGCAAC
CATACCACCAGCAAATGCAAAGTGCAGGTGAACGAAGTGTATGGCTTAGTAGATG
GCTTAGTTACCTTCTATCTGCCATTACTAATTATGTGCATAACCTATTATAGAATCTT
CAAAGTTGCTAGAGATCAGGCTAAGCGCATTAACCATATTAGTAGTTGGAAGGCT
GCTACTATTCGTGAACATAAAGCTACAGTTACCTTAGCAGCAGTAATGGGAGCATT
TATCATTTGCTGGTTTCCGTACTTTACAGCATTTGTATATAGAGGCTTAAGAGGCG
ATGATGCGATTAACGAAGTATTAGAAGCGATTGTGCTGTGGTTAGGCTATGCGAA
CTCTGCGTTGAACCCGATTCTGTATGCTGCTCTGAACCGCGACTTTAGAACTGGC
TATCAGCAGCTATTCTGTTGTCGTTTAGCTAACCGTAACAGTCATAAGACTAGCTT
AAGATCTAACGCGTCACAGCTGAGCCGCACCCAGTCTCGTGAACCTAGACAGCAG
GAAGAGAAACCATTGAAATTACAAGTGTGGTCAGGTACTGAAGTTACTGCTCCTCA
GGGCGCTACTGATAGATAA

>Human Histamine Receptor 2 F254A – HR_{H2} F254A

ATGGCACCTAACGGTACTGCATCTAGCTTCTGCTTAGATTCTACTGCTTGTAAGAT
TACTATTACTGTAGTATTAGCTGTATTGATTCTAATTACCGTAGCTGGCAACGTAGT
AGTATGCTTGGCTGTAGGCTTGAACAGAAGATTGCGCAACTTGACCAACTGCTTTA
TTGTTAGCTTAGCGATTACCGATCTACTACTAGGCCTACTGGTTCTACCGTTTAGC
GCTATTTATCAGTTATCATGCAAATGGAGCTTTGGTAAAGTGTTCTGCAACATTTAT
ACTTCTCTAGATGTGATGTTGTGCACCGCTTCAATTCTGAACCTGTTTATGATTAGC
TTAGATCGCTATTGCGCGGTAATGGACCCATTACGCTATCCAGTGTTAGTGACTCC
TGTTAGAGTTGCGATTAGCCTGGTGCTGATTTGGGTGATTAGCATTACCCTGAGCT
TTCTGAGCATTCATCTGGGATGGAATAGCCGCAACGAAACCAGCAAAGGCAACCA
TACCACCAGCAAATGCAAAGTGCAGGTGAACGAAGTGTATGGCTTAGTAGATGGC

TTAGTTACCTTCTATCTGCCATTACTAATTATGTGCATAACCTATTATAGAATCTTCA
AAGTTGCTAGAGATCAGGCTAAGCGCATTAACCATATTAGTAGTTGGAAGGCTGCT
ACTATTCGTGAACATAAAGCTACAGTTACCTTAGCAGCAGTAATGGGAGCATTTAT
CATTTGCTGGTTTCCGTACTTTACAGCAGCTGTATATAGAGGCTTAAGAGGCGATG
ATGCGATTAACGAAGTATTAGAAGCGATTGTGCTGTGGTTAGGCTATGCGAACTCT
GCGTTGAACCCGATTCTGTATGCTGCTCTGAACCGCGACTTTAGAACTGGCTATC
AGCAGCTATTCTGTTGTCGTTTAGCTAACCGTAACAGTCATAAGACTAGCTTAAGA
TCTAACGCGTCACAGCTGAGCCGCACCCAGTCTCGTGAACCTAGACAGCAGGAA
GAGAAACCATTGAAATTACAAGTGTGGTCAGGTACTGAAGTTACTGCTCCTCAGG
GCGCTACTGATAGATAA

>Human Histamine Receptor 2 D186A – HR$_{H2}$ D186A

ATGGCACCTAACGGTACTGCATCTAGCTTCTGCTTAGATTCTACTGCTTGTAAGAT
TACTATTACTGTAGTATTAGCTGTATTGATTCTAATTACCGTAGCTGGCAACGTAGT
AGTATGCTTGGCTGTAGGCTTGAACAGAAGATTGCGCAACTTGACCAACTGCTTTA
TTGTTAGCTTAGCGATTACCGATCTACTACTAGGCCTACTGGTTCTACCGTTTAGC
GCTATTTATCAGTTATCATGCAAATGGAGCTTTGGTAAAGTGTTCTGCAACATTTAT
ACTTCTCTAGATGTGATGTTGTGCACCGCTTCAATTCTGAACCTGTTTATGATTAGC
TTAGATCGCTATTGCGCGGTAATGGACCCATTACGCTATCCAGTGTTAGTGACTCC
TGTTAGAGTTGCGATTAGCCTGGTGCTGATTTGGGTGATTAGCATTACCCTGAGCT
TTCTGAGCATTCATCTGGGATGGAATAGCCGCAACGAAACCAGCAAAGGCAACCA
TACCACCAGCAAATGCAAAGTGCAGGTGAACGAAGTGTATGGCTTAGTAGCTGGC
TTAGTTACCTTCTATCTGCCATTACTAATTATGTGCATAACCTATTATAGAATCTTCA
AAGTTGCTAGAGATCAGGCTAAGCGCATTAACCATATTAGTAGTTGGAAGGCTGCT
ACTATTCGTGAACATAAAGCTACAGTTACCTTAGCAGCAGTAATGGGAGCATTTAT
CATTTGCTGGTTTCCGTACTTTACAGCATTTGTATATAGAGGCTTAAGAGGCGATG
ATGCGATTAACGAAGTATTAGAAGCGATTGTGCTGTGGTTAGGCTATGCGAACTCT
GCGTTGAACCCGATTCTGTATGCTGCTCTGAACCGCGACTTTAGAACTGGCTATC
AGCAGCTATTCTGTTGTCGTTTAGCTAACCGTAACAGTCATAAGACTAGCTTAAGA
TCTAACGCGTCACAGCTGAGCCGCACCCAGTCTCGTGAACCTAGACAGCAGGAA
GAGAAACCATTGAAATTACAAGTGTGGTCAGGTACTGAAGTTACTGCTCCTCAGG
GCGCTACTGATAGATAA

>Human Histamine Receptor 2 Y250A – HR$_{H2}$ Y250A

ATGGCACCTAACGGTACTGCATCTAGCTTCTGCTTAGATTCTACTGCTTGTAAGAT
TACTATTACTGTAGTATTAGCTGTATTGATTCTAATTACCGTAGCTGGCAACGTAGT
AGTATGCTTGGCTGTAGGCTTGAACAGAAGATTGCGCAACTTGACCAACTGCTTTA
TTGTTAGCTTAGCGATTACCGATCTACTACTAGGCCTACTGGTTCTACCGTTTAGC
GCTATTTATCAGTTATCATGCAAATGGAGCTTTGGTAAAGTGTTCTGCAACATTTAT
ACTTCTCTAGATGTGATGTTGTGCACCGCTTCAATTCTGAACCTGTTTATGATTAGC
TTAGATCGCTATTGCGCGGTAATGGACCCATTACGCTATCCAGTGTTAGTGACTCC
TGTTAGAGTTGCGATTAGCCTGGTGCTGATTTGGGTGATTAGCATTACCCTGAGCT
TTCTGAGCATTCATCTGGGATGGAATAGCCGCAACGAAACCAGCAAAGGCAACCA
TACCACCAGCAAATGCAAAGTGCAGGTGAACGAAGTGTATGGCTTAGTAGATGGC
TTAGTTACCTTCTATCTGCCATTACTAATTATGTGCATAACCTATTATAGAATCTTCA

AAGTTGCTAGAGATCAGGCTAAGCGCATTAACCATATTAGTAGTTGGAAGGCTGCT
ACTATTCGTGAACATAAAGCTACAGTTACCTTAGCAGCAGTAATGGGAGCATTTAT
CATTTGCTGGTTTCCGGCTTTTACAGCATTTGTATATAGAGGCTTAAGAGGCGATG
ATGCGATTAACGAAGTATTAGAAGCGATTGTGCTGTGGTTAGGCTATGCGAACTCT
GCGTTGAACCCGATTCTGTATGCTGCTCTGAACCGCGACTTTAGAACTGGCTATC
AGCAGCTATTCTGTTGTCGTTTAGCTAACCGTAACAGTCATAAGACTAGCTTAAGA
TCTAACGCGTCACAGCTGAGCCGCACCCAGTCTCGTGAACCTAGACAGCAGGAA
GAGAAACCATTGAAATTACAAGTGTGGTCAGGTACTGAAGTTACTGCTCCTCAGG
GCGCTACTGATAGATAA

>Human Histamine Receptor 2 Y182A – HR_{H2} Y182A

ATGGCACCTAACGGTACTGCATCTAGCTTCTGCTTAGATTCTACTGCTTGTAAGAT
TACTATTACTGTAGTATTAGCTGTATTGATTCTAATTACCGTAGCTGGCAACGTAGT
AGTATGCTTGGCTGTAGGCTTGAACAGAAGATTGCGCAACTTGACCAACTGCTTTA
TTGTTAGCTTAGCGATTACCGATCTACTACTAGGCCTACTGGTTCTACCGTTTAGC
GCTATTTATCAGTTATCATGCAAATGGAGCTTTGGTAAAGTGTTCTGCAACATTTAT
ACTTCTCTAGATGTGATGTTGTGCACCGCTTCAATTCTGAACCTGTTTATGATTAGC
TTAGATCGCTATTGCGCGGTAATGGACCCATTACGCTATCCAGTGTTAGTGACTCC
TGTTAGAGTTGCGATTAGCCTGGTGCTGATTTGGGTGATTAGCATTACCCTGAGCT
TTCTGAGCATTCATCTGGGATGGAATAGCCGCAACGAAACCAGCAAAGGCAACCA
TACCACCAGCAAATGCAAAGTGCAGGTGAACGAAGTGGCTGGCTTAGTAGATGGC
TTAGTTACCTTCTATCTGCCATTACTAATTATGTGCATAACCTATTATAGAATCTTCA
AAGTTGCTAGAGATCAGGCTAAGCGCATTAACCATATTAGTAGTTGGAAGGCTGCT
ACTATTCGTGAACATAAAGCTACAGTTACCTTAGCAGCAGTAATGGGAGCATTTAT
CATTTGCTGGTTTCCGTACTTTACAGCATTTGTATATAGAGGCTTAAGAGGCGATG
ATGCGATTAACGAAGTATTAGAAGCGATTGTGCTGTGGTTAGGCTATGCGAACTCT
GCGTTGAACCCGATTCTGTATGCTGCTCTGAACCGCGACTTTAGAACTGGCTATC
AGCAGCTATTCTGTTGTCGTTTAGCTAACCGTAACAGTCATAAGACTAGCTTAAGA
TCTAACGCGTCACAGCTGAGCCGCACCCAGTCTCGTGAACCTAGACAGCAGGAA
GAGAAACCATTGAAATTACAAGTGTGGTCAGGTACTGAAGTTACTGCTCCTCAGG
GCGCTACTGATAGATAA

>Human Histamine Receptor 2 T190A – HR_{H2} T190A

ATGGCACCTAACGGTACTGCATCTAGCTTCTGCTTAGATTCTACTGCTTGTAAGAT
TACTATTACTGTAGTATTAGCTGTATTGATTCTAATTACCGTAGCTGGCAACGTAGT
AGTATGCTTGGCTGTAGGCTTGAACAGAAGATTGCGCAACTTGACCAACTGCTTTA
TTGTTAGCTTAGCGATTACCGATCTACTACTAGGCCTACTGGTTCTACCGTTTAGC
GCTATTTATCAGTTATCATGCAAATGGAGCTTTGGTAAAGTGTTCTGCAACATTTAT
ACTTCTCTAGATGTGATGTTGTGCACCGCTTCAATTCTGAACCTGTTTATGATTAGC
TTAGATCGCTATTGCGCGGTAATGGACCCATTACGCTATCCAGTGTTAGTGACTCC
TGTTAGAGTTGCGATTAGCCTGGTGCTGATTTGGGTGATTAGCATTACCCTGAGCT
TTCTGAGCATTCATCTGGGATGGAATAGCCGCAACGAAACCAGCAAAGGCAACCA
TACCACCAGCAAATGCAAAGTGCAGGTGAACGAAGTGTATGGCTTAGTAGATGGC
TTAGTTGCTTTCTATCTGCCATTACTAATTATGTGCATAACCTATTATAGAATCTTCA

AAGTTGCTAGAGATCAGGCTAAGCGCATTAACCATATTAGTAGTTGGAAGGCTGCT
ACTATTCGTGAACATAAAGCTACAGTTACCTTAGCAGCAGTAATGGGAGCATTTAT
CATTTGCTGGTTTCCGTACTTTACAGCATTTGTATATAGAGGCTTAAGAGGCGATG
ATGCGATTAACGAAGTATTAGAAGCGATTGTGCTGTGGTTAGGCTATGCGAACTCT
GCGTTGAACCCGATTCTGTATGCTGCTCTGAACCGCGACTTTAGAACTGGCTATC
AGCAGCTATTCTGTTGTCGTTTAGCTAACCGTAACAGTCATAAGACTAGCTTAAGA
TCTAACGCGTCACAGCTGAGCCGCACCCAGTCTCGTGAACCTAGACAGCAGGAA
GAGAAACCATTGAAATTACAAGTGTGGTCAGGTACTGAAGTTACTGCTCCTCAGG
GCGCTACTGATAGATAA

References

1. Mukherjee, K.; Bhattacharyya, S.; Peralta-Yahya, P., GPCR-Based Chemical Biosensors for Medium-Chain Fatty Acids. *ACS Synth Biol* **2015**, 4, 1261-1269.
2. Yasi, E. A.; Allen, A. A.; Sugianto, W.; Peralta-Yahya, P., Identification of Three Antimicrobials Activating Serotonin Receptor 4 in Colon Cells. *ACS Synth Biol* **2019**, 8, 2710-2717.
3. Marquez-Gomez, P. L.; Kruyer, N. S.; Eisen, S. L.; Torp, L. R.; Howie, R. L.; Jones, E. V.; France, S.; Peralta-Yahya, P., Discovery of 8-Hydroxyquinoline as a Histamine Receptor 2 Blocker Scaffold. *ACS Synth Biol* **2022**, 11, 2820-2828.
